# Pharmacological and dietary prevention for colorectal cancer

**DOI:** 10.1186/1471-2482-13-S2-S16

**Published:** 2013-10-08

**Authors:** Francesca Nolfo, Stefania Rametta, Stefano Marventano, Giuseppe Grosso, Antonio Mistretta, Filippo Drago, Santi Gangi, Francesco Basile, Antonio Biondi

**Affiliations:** 1 Department "G. F. Ingrassia" Section of Hygiene and Public Health, University of Catania, Catania, Italy; 2 Department of Drug Sciences, Section of Biochemistry, University of Catania, Catania, Italy; 3 Department of Clinical and Molecular Biomedicine, Section of Pharmacology and Biochemistry, University of Catania, Catania, Italy; 4 Department of General Surgery, Section of General Surgery and Oncology, University Medical School of Catania, Italy

## Abstract

**Background:**

Colorectal cancer (CRC) is a leading cause of cancer morbidity and mortality. People at higher risk are those individuals with a family history of CRC and familial adenomatous polyposis. Prevention and screening are two milestones for this disease. The aim of this study is to evaluate the chemopreventive role of non-steroidal anti-inflammatory drugs (NSAIDs), including aspirin and cyclooxygenase 2 inhibitors, some micronutrients (folic acid, calcium, selenium, antioxidants) and probiotics.

**Discussion:**

The studies on aspiring reported promising results, but it is debatable whether aspirin should be used as chemoprevention, because of its side effects and because of poor efficacy evident in subjects at high risk. Similar results were reported for other non-aspirin NSAIDs, such as sulindac and celecoxib, which the potential adverse effects limit their use. Selenium role in prevention of various types of cancer as well as in colon adenomas are often inconclusive or controversial. Several studies suggested that calcium may have a possible chemopreventive effect on colon adenomas and CRC, although contrasting results are reported for the latter. A recent meta-analysis including 13 randomized trial suggested that folic acid supplementation had not a chemiopreventive action on CRC. Several studies investigated the association between antioxidants, administered alone or in combination, and CRC risk, both among general and at risk population, but only few of them supported statistically significant results.

**Conclusion:**

The results of this literature review showed an unclear role in CRC prevention of both pharmacological and dietary intervention. Despite several options are available to prevent colon cancer, it is challenging to identify a correct strategy to prevent CRC through pharmacological and dietary intervention due to the long latency of cancer promotion and development. Since some of the drugs investigated may have uncertain individual effects, it can be suggested to potentiate such effects by adding them together.

## Background

During last decades, several progresses have been done in cancer prevention [1]. The introduction of the laparoscopic approach allowed a less invasive surgical therapy (as tertiary prevention) for colorectal cancer (CRC) patients [2-6]. Regarding the secondary prevention, the screening programs demonstrated high value in certain types of cancer, but only allow preventing progression of already existing malignancies that often occur in elderly subjects [7,8]. Regarding CRC, which is the third most common cancer in males and the second in females [9], special attention has been paid to the adenomatous polyp or adenoma, which is an important surrogate endpoint for colon cancer [10]. The adenoma is a premalignant lesion, usually asymptomatic, which occurs generally among older individuals, especially those more than 50 years old [10]. It is common that colon cancers emerge from adenomas, although not all adenomas evolve in cancers [10,11]. However, since new adenomas often develop in individuals who have had previous adenomas that were ablated, a preventive intervention may focus on prevent formation of new adenomas as a surrogate marker of colon cancer.

Despite the recent development in drug therapy against CRC are promising [12,13], primary prevention interventions against colorectal cancer mostly involve dietary measures, although a pharmacological approach demonstrated to be effective in selected subjects. Thus, the aim of this study is to review the effects of dietary and pharmacological experimental approach to identify chemopreventive agents and strategies against the CRC.

## Aspirin

Acetylsalicylic acid (aspirin) is one of the most widely used drugs in the world [14]. Aspirin is a salicylate drug, nowadays mainly used as an analgesic to calm minor aches and pains, as an antipyretic to reduce fever, and as an anti-inflammatory medication. It also plays an important role in hypertension and in other cardiovascular diseases [15]. In fact, it has been long known that thanks to his antiplatelet effect by inhibiting the production of thromboxane, aspirin is used long-term, at low doses, to help prevent ischemic events [16]. The main undesirable side effects of aspirin assumption are gastrointestinal ulcers and stomach bleeding, especially in higher doses. Nausea and dyspepsia are milder adverse reactions, and fortunately the most frequent [17]. Because of his already mentioned antiplatelet effect, the use of aspirin in association with other drugs that increase the risk of bleeding must be controlled. In addition, aspirin should be used with prudence in the elderly, because of the risk of Reye's syndrome [18] and in individuals with favism, although recent studies have shown a good tolerance to low doses of the drug in these patients [19].

Despite aspirin at present is not a certified medicinal for cancer prevention, several lines of evidence propose that long-term use of aspirin might decrease the risk of some cancers, particularly gastrointestinal tumors [20]. Different researches have observed an inverse association between continuous aspirin intake and risk of CRC [21-23]. In 1991, Thun et al. [24] recruited 662,424 patients, proving a reduced risk of CRC mortality over a 6-year period, thanks to aspirin use at least 16 times per month. Similar findings were observed in subsequent studies, such as the Health Professionals Follow-up Study (HPFS) and the US Nurses' Health Study (NHS) cohort, which talked about an hazard reduction respectively equal to 21% in a cohort of men and 23% in a cohort of women only [25,26]. Two American [27,28] and two European randomized studies [29,30] compared aspirin against a placebo group in the intermediate risk population, consisting of patients with history of adenomas. Sandler et al. [27] showed that daily aspirin intake for one year reduced by approximately 35% the risk of adenoma, of both the number and the rapidity of growth, in patients who had previously undergone surgical resection of colorectal cancer. The other study conducted in USA was a randomized, double-blind, placebo-controlled trial about the efficacy of different doses of oral aspirin, comparing 81 mg and 325 mg of aspirin per day with placebo in patients with a recent history of adenoma and finding that the relative risk (RR) of adenoma reappearance was lower in the 81 mg group than in the 325 mg one [28]. The same result about the effectiveness of aspirin in lowering the recurrence of colorectal adenomas in intermediated risk population has been demonstrated by Logan et al. [29], which also underlined a possible role of the drug in preventing the development of advanced lesions. In this 3-year study, folic acid was prescribed in addiction to aspirin to half of the patients in both the treated and the control group, but the evidence showed no protective efficacy conferred by this other medicine. Finally, in a study of 238 patients, with a history of adenoma, randomized to receive placebo, 160 mg aspirin, or 300 mg aspirin, the RR of adenoma recurrence was 0.61 in the 300 mg group, and 0.85 in the 160 mg group [30]. Besides, the RR for recurrent adenomas for the 300-mg group versus the 160 mg group was 0.72.

Although these promising result, it is debatable whether aspirin should be used as chemoprevention, because of its side effects and because of poor efficacy evident in subjects at high risk. Some studies specifically investigated the association between the use of aspirin and the risk of intestinal neoplasms among high risk population, such as patients with hereditary conditions with a known genetic basis, for instance adults with Lynch syndrome) [31] and people with familial adenomatous polyposis (FAP) [32]. Both studies compared aspirin (600 mg daily), with or without resistant starch (30 g daily), to resistant starch alone or placebo, demonstrating no benefits in reducing polyp number or the incidence of colorectal cancer. Nevertheless, the Colorectal Adenoma/carcinoma Prevention Program 1 (CAPP1) study founded a significant decline in polyp size among patients with FAP treated with aspirin for more than 1 year [32]. A probable different mismatch-repair-dependent neoplastic pathway, characterized by low susceptibility to protection from aspirin, may explain the absent efficacy of this drug in high-risk patients compared with those at intermediate risk [31]. Finally, several researches that analyzed the effect of aspirin on adenomas and CRC in low-risk populations have found that alternate day use of low-dose aspirin for an average 10 or 5 years of treatment did not lower CRC risk [33,34]. Other authors, instead, studied the effects of daily administered high dose of aspirin (300-500 mg/day for 1-7 years and 300-1500 mg/day for 5-6 years) but still no inverse relationship between aspirin use and risk of adenomas or CRC was found [35,36]. On the other hand, an updated analysis of these cohorts made by Flossmann et al. [37], observed that over a follow-up period of up to 23 years there was a statistically significant 26% reduction in the relative risk of CRC incidence in aspirin users versus nonusers.

Regarding the possible mechanism of action of aspirin in preventing oncological diseases, it may depends on its most well-known pharmacologic effect such as the permanent modification of the COX enzymes, which are responsible for the conversion of arachidonic acid to prostaglandins, relating eicosanoids. Cyclo-oxygenase-1 is in many tissues, while the isoenzyme 2 of COX is almost undetectable in normal tissue and produces prostaglandins for inflammatory response. COX-2 is up-regulated in many cancers such as CRC but also in colorectal adenomas [38,39]. In fact, as suggested by different studies COX-2 may play a role in colorectal tumor growth and development, by its effect on pro- and anti-inflammatory cytokines, migration, apoptosis transcription, and angiogenic factors [40,41]. It seems that the chemopreventive effect, still unclear, of aspirin against CRC is due to the COX-2 inhibitory action both directly, by suppressing prostanoids release, and indirectly, through its effects on platelets [38]. In fact, aspirin mediated inhibition of platelets may block the liberation of mediators that promote COX-2 expression in nucleated cells at sites of mucosal injury [39]. There are also some non-COX-related pathways that may explain aspirin's antineoplastic effects, such as the promotion of apoptosis in cancer cells [42], the diminution of microsatellite instability in CRC cells [43], the modulation of expression of transcription factors induced by oncogenes and the induction of spermidine/spermine N1-acetyltransferase, which results in modulation of polyamine synthesis, essential for neoplastic cell growth [44,45].

## Non-aspirin non-steroidal anti-inflammatory drugs (NSAIDs)

Study on chemoprevention of CRC and/or adenomatous polyps in populations at differing risks investigated the role of NSAIDs, including aspirin and cyclo-oxygenase-2 (COX-2) inhibitors. The only drug authorized for this purpose by the European Medicines Agency is the Celecoxib a non-aspirin NSAIDs selectively blocker COX-2. It was demonstrated to be effective in reducing the number of polyps in patients with familial adenomatous polyposis (FAP) and it is also used in addition to surgery and endoscopic surveillance [46]. Many controlled trials have been made in different countries to investigate NSAIDs efficacy and safety in adenomas and cancer chemoprevention. A randomized, double-blind, placebo-controlled study on 41 young subjects (8-25 years) genotypically, but not phenotypically, affected with FAP were treated with sulindac or placebo for 48 months. The results showed that standard doses of sulindac (75 or 150 mg twice a day) did not prevent the development of adenomas in subjects with FAP [47]. A previous trial conducted on 22 patients with FAP receiving sulindac at the dose of 150 mg orally twice a day for nine months showed a reduction in numbers and size of colorectal adenomas. However, after one year of follow-up, this treatment demonstrated only a partial efficacy, and cannot replace colectomy as primary therapy [48]. Nugent et al. [49] conducted a randomized trial on 24 patients with FAP and advanced duodenal polyposis who had previously undergone prophylactic colectomy. Patients received sulindac at the dose of 400 mg once a day for six months. At the end of the treatment period a significant regression both in duodenal and rectal polyps was found.

The effectiveness of sulindac in inducing the regression of rectal polyps in FAP was also demonstrated by another double-blind trial conducted on 10 patients with rectal polyps and previously treated by colectomy and ileorectal anastomosis [50]. Patients received sulindac (300 mg/day) or placebo during two 4-month periods separated by a 1-month washout phase. The authors observed significant differences in complete and almost complete regression between the sulinac and the placebo groups. Other placebo-controlled, double-blind trials, showed a significant regression of colorectal adenomas in patients with FAP using 400 mg of celecoxib twice daily [51] and a significantly reduction of recurrence of colorectal adenomas within three years after polypectomy using 400 mg of celecoxib once daily [52]. On the contrary, another study showed that the inhibition of a COX-2 with a 150-200 mg dose of a selective COX-2 inhibitor did not induce clinically sufficient regression of adenomas in patients with FAP in a limited (6-month) medication period [53].

Despite the positive results of the use of NSAIDs in chemoprevention of CRC, the potential adverse effects limit their use. Results from a trial conducted on 2.035 subjects that received 200 mg or 400 mg of calecoxib twice daily for 3 years, found a reduction in the incidence of colorectal adenomas, compared with the placebo one, but also an increased risk of cardiovascular events. The RR was 2.6 (95% CI 1.1-1.6) and 3.4 (95% CI 1.5-7.9) for low and high dose respectively [54]. These results are in according with a recent double-blind trial on 1561 subjects with removed colorectal adenomas followed for 5 years. At the end of the follow up the incidence of new and advanced adenomas were significantly lower in the celecoxib group than in the placebo group, however renal/hypertension events (RR 1.35; 95% CI 1.09-1.68) and cardiac disorders (RR 1.59; 95% CI 1.12-2.26) were highter in the celecoxib group [55].

A more recent study focused on the effect of NSAIDs on various pro- and anti-inflammatory cytokines, transcription, and angiogenic factors that are involved in the promotion and growing of many cancers. The findings suggest that co-administration of Sulindac and Celecoxib significantly reduce the angiogenic potential of the growing neoplasm, but its consumption is not toxic free (serious adverse events to gastrointestinal, renal and cardiovascular systems have been reported) [56]. A possible solution for the adverse effect of NSAIDs used as treatment of FAP could be the administration of colon-specific delivery of these drugs. Lee et al. [57] has found that the use of colon-specific prodrug of celecoxib may be a useful strategy to reduce the toxic effect and improve the pharmacological properties of celecoxib.

## Selenium

Selenium is an essential micronutrient present in nature implicated in numerous biological functions, such as a protective effect against oxidative damage of cells, formation of thyroid hormones and modulation of immune system [58,59]. Most of the selenium in the body comes from diet. The aliments richest in selenium are liver, fish, poultry and wheat. The recommended daily intake of selenium varies from 60 μg/day for women, to 70 μg/day for men [59]. Selenium role in prevention of various types of cancer as well as in colon adenomas are often inconclusive or controversial. An experimental study attributed the potential preventive action of selenium to its role in the acceleration of DNA repair and in the reduction of DNA damage, with a decrease in number of mutations that ultimately contribute to carcinogenesis [60]. Another possible mechanisms involved could be the induction of apoptosis through the activation of caspase-3 and the modulation of glutathione and mitochondrial functions [61].

Clark et al. [62] in a multicenter, double-blind, randomized, placebo-controlled cancer prevention trial conducted on 1312 subject with a history of basal cell or squamous cell carcinomas of the skin (receiving 200 µg of selenium or placebo per day) showed that the daily administration of selenium had no protective effect against the development of skin cancer. Nevertheless, a significant reductions in total cancer mortality in selenium treatment group (RR 0.50; 95% CI 0.31-0.80) and in total cancer incidence (RR 0.63; 95% CI 0.47-0.85) was found. Conversely other studies didn't found a protective role of selenium in cancer [63-65].

A meta-analysis on antioxidant supplements for prevention of gastrointestinal cancers confirmed the no role of selenium in cancer prevention with a RR of 0.48 (95% CI 0.22-1.05) [66]. However in four of the fourteen trials evaluated, selenium showed significant beneficial effect on the incidence of gastrointestinal cancer (RR 0.50; 95% CI 0.35-0.71). The authors pointed out that this result might be biased because of low methodological quality in three of four trials.

On the preventive effect of colorectal adenoma selenium showed a significant effect in specific group of subject. An inverse association between serum selenium and advanced colorectal adenoma was found in current smoker (OR 0.27; 95% CI 0.11-0.66) and in subject with the lowest tertile of baseline selenium (OR 0.27; 95% CI 0.09-0.77) [67]. These results are also confirmed by another randomized trial on 758 cases and 767 sex- and race-matched controls, where an OR of 0.63 (95% CI 0.47-0.84) and 0.44 (95% CI 0.24-0.81) were found for the lowest quintile of baseline selenium and current smokers, respectively [68]. Finally a more recent double-blind randomized trial conducted on 411 patients who had undergone a polypectomy, showing that the co-administration of selenium with other antioxidants significantly reduced the risk of adenoma recurrence in these patients (HR 0.61; 95 % CI 0.41-0.92) [69].

## Calcium

Calcium is essential for living organisms being involved in biological processes and being one of the main components of bones, teeth and nails. It is not currently a recognized chemopreventive agent for colon cancer but its intake is recommended in cases of deficient such as osteoporosis or in older adults. The use must be controlled and limited in cases of kidney failure, sarcoidosis or nephrolithiasis and its administration is contraindicated in the patients with hypercalcaemia and hypercalciuria. In case of excess can cause gastrointestinal disturbances, bradycardia and arrhythmias [70].

The mechanisms of action of calcium implicated in the inhibition of colorectal carcinogenesis include the control of cell proliferation, differentiation, and apoptosis. Numerous input signals and intracellular pathways are responsive to calcium and the response of a cell is strictly contextual and linked to the cell type [71]. The modulation of calcium-sensing receptors with the following cascade of intracellular events and the direct role as activator/cofactor in protein kinase C activation are only two of the essential role of calcium in the cell growth and differentiation. Furthermore calcium is also involved in the transcription process through the direct influence of CREB (cAMP response element-binding protein) [72]. In particular, colonocytes respond to calcium exposure with the acquisition of a differentiated phenotype and their sensibility to calcium remain until the disruption of the calcium signaling differentiation-inducing network by the neoplastic process [73]. There is also some evidence on normal and transformed colonic cells of the direct anti-proliferative, differentiation and apoptosis-inducing action of calcium [74,75].

Several studies suggested that calcium may have a possible chemopreventive effect on colon adenomas and CRC. A recent analysis conducted on 1.235 subjects (420 with CRC and 815 controls) investigated the dietary calcium intake and its possible interactions with calcium-sensing receptor (CASR) gene polymorphisms on colorectal cancer risk. A food frequency questionnaire and single nucleotide polymorphisms (SNPs) of CASR research were performed and in all of the polymorphisms examined a significant higher odds ratios was found in the low-calcium-intake group compared to the high-calcium-intake group [76]. In subject with a history of adenoma many randomize trial found a slightly effect of calcium. Thomas et al. [77] showed that calcium reduces colorectal cell turnover but no effect on the number, size or distribution of rectal polyps in a double-blind study carried out on 25 patients with FAP receiving supplemental calcium carbonate (1.5 g/day) or placebo tablets for 6 months. Another study, performed on patients that received calcium gluconolactate and carbonate (2 g elemental calcium daily) or placebo for 3 years, showed a modest but not significant reduction in the risk of adenoma recurrence (OR 0.66 95% CI 0.38-1.17) [78] On the contrary, a trial performed on 930 subject receiving 1.2 g of elementary calcium daily for 4 years, showed that the calcium group, compared with the placebo one, had a lower risk to have at least one adenoma (OR 0.81; 95% CI 0.67-0.99) and also in the average number of adenomas (OR 0.76; 95% CI 0.60 to 0.96) [79]. In a prospective intervention study, 116 polyp-bearing patients received 1.6 g/day of calcium combined with some antioxidants (beta-carotene, vitamin E, vitamin C, selenium) were followed for 3 years. At 3 years no differences were found on polyp growth between the intervention and the placebo group. However a significant reduction in the number of new adenomas was found, suggesting a possible protective role of calcium in the formation of new adenomas [80]. A most recent study assessing the predictive value of CRC risk factors for the detection of advanced colorectal adenoma on 1236 randomly selected subject, confirmed that calcium intake could have a small protective effect (OR 0.99 per mg; 95% CI 0.99-1.00) [81].

Regarding the CRC, a published meta-analysis on sixteen trials investigated the effect of calcium supplements without co-administered vitamin D on cancer risk finding that calcium did not alter the risk of colorectal cancer (RR 1.38; 95% CI 0.89-2.15) [82]. Conversely, another study on 85,903 men and 105,108 women examined the associations of intakes of calcium and vitamin D with CRC risk reporting an inverse association with CRC both for calcium intake [men (RR 0.70; 95% CI 0.52-0.93; p for trend <0.05); woman (RR 0.64; 95% CI 0.50-0.83; p for trend <0.05)] and dairy products [men (RR 0.77; 95% CI 0.59-1.01; p for trend <0.05); women (RR 0.66; 95% CI 0.49- 0.89; p for trend <0.05) [83]. As the previous study, other authors investigated the associations between the consumption of dairy foods and calcium with cancer risk. One of these studies conducted on 36,965 men and 16,605 women cancer cases failed in found a linear association between calcium intake and cancer [84]. However dairy food and calcium intakes were significantly associated with digestive system cancer (RR 0.84; 95% CI 0.77-0.92 in men, and RR 0.77; 95% CI 0.69-0.91 in women) and particularly with CRC both in men and women. Cho et al. [85] analyzed a pool of 10 cohort studies included 534,536 individuals, among whom 4,992 incident cases of colorectal cancer. Compared with the lowest intake category of milk, dietary calcium and total calcium the RR for the highest category were 0.85 (95% CI 0.78-0.94; p for trend <0.05), 0.86 (95% CI 0.78-0.95; p for trend <0.05) and 0.78 (95% CI 0.69- 0.88; p for trend <0.001), respectively. These results are also confirmed by a meta-analysis conducted to evaluate the relationship between the consumption of dairy products, calcium, vitamin D and the inhibition of the development of CRC [86]. Data from 60 epidemiological studies enrolling 26.335 CRC cases were analyzed. High milk and dairy product intake RR on colon cancer risk were, respectively, 0.78 (95% CI 0.67-0.92) and 0.84 (95% CI 0.75-0.95). Furthermore a high calcium intake was found to reduce CRC risk.

Other studies investigated the role of calcium or calcium plus vitamin D in CRC prevention focusing their research on population subgroups. The trial reported by Lappe et al. [87] was conducted on 1,179 postmenopausal women divided in three groups: calcium alone (1.4-1.5 g/day), calcium (1.4-1.5 g/day) plus vitamin D (1100 IU) and placebo. The treatment period was four years as well as the follow up period. The results showed that calcium plus vitamin D (RR 0.40; 95% CI 0.20-0.82) but not calcium alone (RR 0.53; 95% CI 0.27-1.03) reduce all-cancer risk in these subject. On contrary the Wactawski et al. [88] study performed on 36,282 postmenopausal women received calcium 1 g/day plus vitamin D 400 IU/day or placebo for 7 years found no significant effect of daily supplementation of calcium with vitamin D on colorectal cancer incidence. Another study on women with a prospective cohort of 45,354 subjects without a history of colorectal cancer followed for 8.5 years found an approximately 25% reductions CRC risk for both high dietary and supplement calcium intake [89]. Moreover a mayor protective effect was showed for the simultaneously high consumption of calcium, both for dietary and supplement (RR 0.54; 95% CI 0.37-0.79).

## Folic acid

Folic acid is a water-soluble B vitamin needed for all the reactions of synthesis, repair and methylation of DNA, homocysteine metabolism (methylation) and other important biochemical reactions [90]. Humans cannot synthesize folate de novo and the recommended dietary intake change with age, sex or other conditions such as pregnancy or lactation. Therapy with folic acid supplement is indicated in pregnancy to reduce the risk of anemia and to prevent the occurrence of neural tube defects in the fetus [91]. It is also indicated, in combination with vitamin B12, in cases of megaloblastic anemia caused by insufficient dietary intake, and also for the anemia prophylaxis in chronic haemolytic states, malabsorption, or in renal dialysis. Important adverse effects due to folic acid supplementation are very rare, except for some gastrointestinal disorder.

A diet low in folate could be implicated in the onset of many cancers due to folic acid role in stability of DNA by regulating DNA biosynthesis, repair and methylation [92]. In fact, the altered DNA and RNA methylation, and the disruption of DNA integrity and repair, lead to DNA aberrations with a higher risk of developing cancers and in particularly the colorectal one [93]. Furthermore, the results of a randomized trial conducted on fourteen subjects with adenoma receiving 400 mcg/day or placebo for 10 weeks, indicated that folic acid can modify gene expression [94].

The association between diminished folate status or folate supplement and colorectal carcinogenesis had been investigated in many studies. Baron et al. [95] to study the protective effect of fruits and vegetables against the risk of CRC administered a food-frequency questionnaire to patients with adenoma, at baseline and four years later. The obtained results showed a protective effect of folic acid against CRC, however after adjustment for intake of dietary fiber and fat, folate supplements was not associated with a reduction in CRC risk. Furthermore the alcohol intake that can reduce circulating folate levels and interfere with some of its biochemical actions was investigated. Findings reported a significant association between alcohol intake and increased risk of adenoma recurrence (OR 2.04; 95% CI 1.28-3.26) both for man and woman. Similarly another study showed the same results for high alcohol intake, but found more consistent results on the protective effect of high dietary folate, in women RR 0.66 (95% CI 0.46-0.95) and men RR 0.63 (95% CI 0.41-0.98) [96]. On the contrary, a randomized study on women with high risk of cardiovascular disease, receiving folic acid 2.5 mg/day, vitamin B6 50 mg/day and vitamin B12 1 mg/day or placebo for 7 years, showed that the combined intake of these components did not have a significant effect on the risk of cancer occurrence ( HR 0.97; 95% CI 0.79-1.18) [97].

In a double-blind, placebo-controlled trial on 137 subjects with adenomatous polyps, patients received 5 mg/day of folic acid for 3 years showed a significant reduction in the recurrence of colonic adenomas compared to the placebo group [98]. Another trial conducted on 216 patients with atrophic gastritis receiving different combination of folic acid and beta-carotene, demonstrated a lower risk of gastrointestinal cancers in the folic acid group (OR 0.12; 95% CI 0.03-0.51) [99]. Different findings were found by Logan et al. [29] in a multicenter, randomized, double-blind trial on 945 adenoma patients, in which a supplementation of 0.5 mg/day of folic acid for 3 years showed no effect on adenoma recurrence (RR 1.07; 95% CI 0.85-1.34). The same result were showed by a double blind placebo controlled trial conducted on patients with a recent history of colorectal adenomas who received folic acid (1 mg/day) with or without aspirin (81-325 mg/day) for three years. Both at three and five years follow up folic acid was not found to reduce colorectal adenomas [100]. Finally, a recent meta-analysis including 13 randomized trial confirmed these findings, suggesting that folic acid supplementation had not a chemiopreventive action on CRC (RR 1.01; 95% CI 0.82-1.23) [101].

## Probiotics

According to the official definition of FAO and WHO, probiotics are "live organisms which, when administered in adequate amounts, bring benefit to the health of the host" [102]. The mechanisms by which probiotics may inhibit colon cancer are not yet fully understood. However, there is evidence for a reduction in the inflammatory response to host flora, alterations in the metabolic activities of intestinal bacteria, a reduction in the numbers of bacteria involved in pro-carcinogenic and mutagenic pathways, and the production of anti-tumorigenic and mutagenic substances [103]. In an experimental study, Ma et al. [104] showed that the probiotic Bacillus polyfermenticus, through the reduction of ErbB2 and ErbB3 receptors (known to have essential roles in tumor development), suppressed colon cancer cells growth both *in vitro *and *in vivo*. Another study conducted on animal models showed that the intake of probiotics is associated with anticarcinogenic effects, due to the reduction of genotoxic agents in the intestine and, concurrently to the rice in the production of agents that deactivate these toxic components [105]. Rafter et al. [106] conducted a study on 37 colon cancer patients and 43 polypectomized patients that received a synbiotic food composed of prebiotic and probiotics or placebo for twelve weeks. Results showed that the intake of this compound led to positive changes in the bacterial flora and reduced the colorectal proliferation in adenoma patient, but no in the cancer one. Similar results on microflora changes were found in a double bind, placebo controlled study conducted on 20 subjects received resistant starch or Bifidobacterium lactis, either alone or as a combined symbiotic preparation for four weeks. However, no significantly variation on epithelial proliferation was observed [107].

Although many studies investigated the role of probiotics, there is still no clear clinical evidence of the protective role of probiotics in CRC. Further research is needed to establish the real usefulness of probiotics in CRC prevention [108].

## Antioxidants

Antioxidants have been universally studied for prevention of chronic disease, including inflammation, atherosclerosis, neurodegenerative diseases, and also cancer [109-111]. A body of evidence suggests that oxidative stress and the consequent peroxidation of lipid are involved in these pathological states. Antioxidants, such as various vitamins, may be introduced by "eating good", this means following a diet high in fruits and vegetables or as dietary supplements. In fact it was seen that intake of this kind of foods decrease the risk of developing cancer and cardiovascular disease [112].

Vitamins may be prescribed to prevent or treat their corresponding deficit. Lack of vitamin C is a very rare eventuality among people who eat a balanced diet. When it occurs, it causes scurvy, which can lead to death if not treated [109]. Also vitamin A (retinol) deficiency is uncommon and it is correlated with ocular problems, such as night blindness or xerophthalmia, and immune system disorders. On the other hand, excessive doses of the vitamin may be toxic, causing, among other things, birth defects. Thus, retinol additions are contraindicated specifically in pregnant women. Some studies reported the necessity of vitamin E intake in adulthood, especially if a possible cholestasis causes fats malabsorption. It seems that this medicament could play a role in the treatment of thromboembolic disease even if its use should be done with prudent. As the previous one, an excess of vitamin E (> 1 g/day) can produce side effects, such as diarrhea and abdominal pain [113]. Finally, another important antioxidant is vitamin D, which consists of ergocalciferol, or vitamin D2, colecalciferol, also known as vitamin D3, dihydrotachysterol, alfacalcidol and calcitriol. Vitamin D2 is present in plants and some fish, so that its deficiency results from a low dietary intake. Vitamin D3 is produced in the skin by way of ultraviolet B rays, thus a low exposure to sunlight can cause its deficiency [114]. Several studies suggest that combined calcium and vitamin D supplementation, but not limited to vitamin D, prevented bone loss and it is recommended to prevent and treat osteoporosis and in elderly patients [115]. Some observational studies in humans and animal models support that vitamin D has a favorable role in cancer prevention, probably due to its action in the regulation of cell growth and differentiation [116].

Focusing on CRC, observational analyses have found an inverse correlation between calcium and vitamin D intake and risk of CRC [78] and recurrent polyps [80].

Thomas et al. [77], considering a high-risk population made by patients with familial adenomatous polyposis (FAP) who had previously undergone total abdominal colectomy with ileorectal anastomosis, observed that calcium had no evident effect on the number, volume or distribution of rectal polyps. Instead, there are no studies about the relationship between antioxidant and colorectal cancer risk in high risk group.

Considering people with previously adenomas or CRC, it has been compared antioxidants, alone or in combination, against placebo. For example, Greenberg et al. [117] selected 864 patients with an history of adenoma removed from large bowel, and dividing them into different treatment groups, administered placebo, beta carotene (25 mg daily), vitamin C (1 g daily) and vitamin E (400 mg daily), or beta carotene plus vitamins C and E. The result of this investigation has demonstrated a lack of chemopreventive action of antioxidants against CRC.

A Canadian study examined one hundred thirty-seven people to value the effects of vitamins C and E against placebo on the rate of recurrence of colorectal polyps, but the correlation found is small and may be attributed to chance (relative risk of 0.86) [118]. A combination of vitamin A, C and E or lactulose were used to evaluate their chemopreventive attitude against the reappearance of colorectal polyps after their endoscopic removal, with positive results. In fact, polyps recurred in 5.7% of the individuals treated with vitamins, 14.7% of patients with lactulose and 35.9% of untreated controls [119]. Similar findings were obtained in an Italian work on 255 individuals after polipectomy [120].

MacLennan et al. [121] performed a randomized, partially double-blinded, placebo-controlled trial to estimate the efficacy on adenomas occurrence of lowering dietary fat to 25% of total calories and fortifying diet with 25 g of wheat bran daily and a capsule of beta carotene (20 mg daily). This found that there was no statistically significant prevention of new adenomas with any of the supplements, although persons who received combined intervention of low fat and added wheat bran developed no adenomas.

Several studies specifically investigated the association between antioxidants, administered alone or in combination, and CRC risk among general population [63,99,122-131]. The average duration of treatment was about 7 years and the follow-up of participants ranged from 5 to 12 years. Among the 14 analyses executed, only few of them supported statistically significant results, such as the one made by Malila et al. [128]. In this work, participants, middle-aged male smokers, were randomly assigned to four supplementation groups: alpha-tocopherol (AT), 50 mg/day; beta-carotene (BC), 20 mg/day; both AT and BC; and placebo. They found an increased CRC risk in the group with α-tocopherol supplementation compared with the no-α-tocopherol group. Instead β-carotene supplementation had no effect on the incidence of adenomas.

However, according to the results from previous reviews, there is no convincing evidence that antioxidant supplements play a significant beneficial role in preventing adenoma or gastrointestinal cancer, including CRC [122,132,133].

## Conclusions

The results of this literature review showed an unclear role in CRC prevention of both pharmacological and dietary intervention. Despite several options are available to prevent colon cancer, it is challenging to identify a correct strategy to prevent CRC through pharmacological and dietary intervention due to the long latency of cancer promotion and development. Since some of the drugs investigated may have uncertain individual effects, it can be suggested to potentiate such effects by adding them together.

## Competing interests

The authors declare that they have no competing interests.

## Authors' contributions

FN: conception and design, drafting the manuscript; SR, GG, AM, SM: drafting the manuscript; FD, FB, AB: critical revision, given final approval of the version to be published

## Declarations

Funding for this article came from University funds.

This article has been published as part of *BMC Surgery *Volume 13 Supplement 2, 2013: Proceedings from the 26th National Congress of the Italian Society of Geriatric Surgery. The full contents of the supplement are available online at http://www.biomedcentral.com/bmcsurg/supplements/13/S2
